# Prevalence and severity of anemia among school children in Jimma Town, Southwest Ethiopia

**DOI:** 10.1186/2052-1839-14-3

**Published:** 2014-01-16

**Authors:** Selomon Assefa, Andualem Mossie, Leja Hamza

**Affiliations:** 1 Lecturer (MSc, Physiologist), Department of Medicine, College of Medicine and Health Sciences, Ambo University, Ambo, Ethiopia; 2 Associate Professor (PhD, Physiologist), Department of Biomedical Sciences, College of Public Health and Medical Sciences, Jimma University, Jimma, Ethiopia; 3 Assistant Professor (MD, Internist), Department of Internal Medicine, College of Public Health and Medical Sciences, Jimma University, Jimma, Ethiopia

**Keywords:** Hemoglobin, Anemia, School children, Prevalence

## Abstract

**Background:**

Anemia is a major health problem worldwide. Because of health and socioeconomic problems, the prevalence of anemia is higher in developing countries. Children and pregnant women are the most vulnerable groups to anemia. The aim of the present study was to determine the magnitude of anemia among school children.

**Methods:**

A cross-sectional household survey was conducted in January 2011 on 423 children, aged 6–14 years, selected through systematic random sampling method. Sociodemographic and anthropometric data were collected using a pre-tested questionnaire. Capillary blood was taken from the fingertip of each child and hemoglobin was measured using HaemoCue digital photometer. All the necessary safety measures were taken during blood collection. Anthropometric indicators were measured using WHO’s guideline. Data analysis was made using SPSS Version 16.0 for Windows. The association between predictors and outcome variables were measured by a stepwise logistic regression model. Ethical permission was obtained; consent of the parents/guardian was taken and confidentiality was maintained.

**Result:**

A total of 404 children were studied. The mean age was 10.21(SD ± 1.89) years. The proportion of females was 217(53.7%). The mean hemoglobin level for both sexes was 11.59(SD ± 1.97 g/dl). The current prevalence of anemia was 152(37.6%), out of which, 73(18.1%) had mild while 79(19.6%) of them had moderate anemia. The prevalence of anemia among the age group of 6–11 years was 118(40.5%) while the prevalence among the group of 12–14 years old children was 34(30.1%). Among the selected variables in the logistic regression analysis, low family income [OR = 4.925, 95% CI(1.063,22.820)], mothers’ education [OR = 4.621, 95% CI(1.383,15.439)], intake of plant food [OR = 3.847, 95% CI(2.068, 7.157)] and intake of animal food [OR = 2.37, 95% CI(1.040,5.402)] were significantly and independently associated with anemia.

**Conclusion:**

Anemia is a moderate public health problem in the study area. Family income, educational status of parents and inadequate plant and animal food intake are the predictors of anemia. Improving the economic status of the family, women education and health education about balanced animal and plant food consumption are recommended strategies to reduce the burden of anemia.

## Background

‘Anemia’ refers to a condition in which the hemoglobin content of the blood is lower than normal as a result of deficiency of one or more essential nutrients [1], heavy blood loss, parasitic infections and congenital hemolytic diseases [2].

Globally, anemia is a public health problem affecting people in both developed and developing countries with bad consequences of human health as well as social and economic development [3,4]. Anemia is a critical health concern because it affects growth and energy levels adversely [2]. It damages immune mechanisms and is also associated with increased morbidity [3]. It occurs at all age groups, but is more prevalent in pregnant women and children [2]. Especially, young children from low income families have a higher risk for developing anemia due to iron deficiency that occurs as a result of high demand for iron during the period of rapid growth [5].

Globally, anemia affects 1.62 billion (24.8%) of the population [4], and an estimated 36% of developing world’s population suffers from this disease [6]. Anemia is known to be a significant global problem affecting 305 million (25.4%) school age children (SAC) [4].

In developing countries, the prevalence of anemia among school age children is 40%, and it is classified as severe public health problem [2,4]. The problem is alarming in Sub-Saharan African Countries such as Kenya 48.9% [7]; Mali 55.8% [8] and Tanzania 79.6% [9]. Lack of awareness among the mothers about the problem coupled with their low educational status [10], poor nutritional practices and unhealthy food habits [11], low iron bioavailability of the diet [12], decreased physical activities [13], malaria and parasitic infestations are additional factors associated with lower hemoglobin (Hb) level in children [14].

Although anemia remains a widespread public health problem in most developing countries, and even developed countries, there are very few studies on the prevalence and severity of anemia among school age children in Jimma area, Ethiopia. Because of its impact on cognitive development and physical growth, studies on the magnitude of anemia among school age children have paramount importance. Anemia creates long term effects among female children resulting in low birthweight babies and post partum hemorrhage. Therefore, the main aim of the present study was to determine the prevalence and severity of anemia on school children.

## Methods

### Study design

A cross-sectional community-based study was conducted among children aged 6 to 14 years in January 2011 in Jimma Town, 354 kilometers southwest of Addis Ababa, Ethiopia. According to the 2007 population and housing census of Ethiopia, the projected total population of Jimma Town in 2010 was 132,052 (Men = 66,343 and Women = 65,708). According to the Office of Education in Jimma Town, there are a total of 24,993 school age children in the town [15]. The sample size of 423 was determined using single population proportion formula. To maximize the sample size, prevalence of anemia (50%) was considered. Ninety five percent certainty and 5% margin of error was taken. Ten percent non-response rate was added to the sample size as a contingency.

### Sampling technique

Systematic random sampling technique was used to select the study participants. Four kebeles were selected randomly out of a total of 13 kebeles of Jimma Town. Kebele is the smallest administrative unit in the governmental structure in Ethiopia. The number of samples taken from each kebele was allocated proportionally. From the selected four kebeles, every 8th household was included in the survey. To start the interview, the first household was selected based on house numbering sequence. One child aged from 6 to 14 years was included from each selected household. If there were more than one child in a household, one was taken for an interview by a lottery method.

### Method of data collection

Sociodemographic data were collected using pre-tested structured questionnaire. Dietary variables were collected using a simplified food frequency questionnaire (FFQ), which is the modified version of Helen Keller International FFQ. This questionnaire has been adopted in Ethiopia, to estimate consumption of foods from animal and plant sources [16].

### Blood test

Portable hemoglobinometer (HemoCue AB, Angelhom, Sweden) was used to determine hemoglobin concentration from a capillary blood sample collected from the fingertip of each child aseptically, using sterile single-use disposable lancet. It was done by trained and experienced laboratory technicians. The necessary safety measures were taken during blood collection. Seventy percent ethanol alcohol was used as disinfectant.

### Anthropometric measurements

Measurements of height and weight were taken according to the WHO’s guideline [17]. Important anthropometric indicators were height-for-age z-scores (HAZ) and BMI z-scores (BMIZ). Weight-for-age z-scores is not a good indicator for children age above 10 years. Because of the pubertal growth surge in children above 10 years, weight-for-age was assessed by BMI-for-age [18].

### Methods of data analysis

Statistical package for the Social Science (SPSS) Version 16.0 for Windows was used for data analysis. Chi-square test and stepwise logistic regression model analysis were carried out to assess the association between independent and outcome variables. P ≤ 0.05 was taken as a minimum level of significance.

### Operational definitions

Anemia was defined in accordance with the WHO standard for children. Accordingly, anemia is defined as Hb level below 12 g/dL in children of 12–14 years and below 11.5 g/dL in children aged 6–11 years [2]. Severe anemia is defined as Hb level below 7 g/dL in children of 6–14 years of age. Moderate anemia is defined as Hb level 7 g/dL - 9.9 gm/dL in children of 6–14 years of age. Mild anemia is defined as Hb level 10 g/dL -11.4 g/dL in children aged 6–11 years and Hb level 10g/dL - 11.9 g/dL in children of 12–14 years of age.

Underweight: z-scores < -2.00 of the WHO median reference for weight-for age. Thinness: z-scores < -2.00 of the WHO median reference for body mass index-for age. Stunting: z-scores < -2.00 of the WHO median reference for height-for-age.

The age group of the children was dichotomized as 6–11 year and 12–14 years [19,20].

### Ethical considerations

Ethical clearance was obtained from the Ethical Board of the Jimma University. Consent was taken from parents/guardians of the children and confidentiality was maintained.

## Results

### Sociodemographic and anthropometric characteristics of the study participants

A total of 423 children were selected, among whom complete response of the anthropometric measurements and blood samples were obtained from 404 students with the response rate of 95.6%. The mean ± SD age was 10.21 ± 1.89 years ranged between 6 and 14 years. From the total of 404 respondents, 291(72%) children were 6–11 years old and 217(53.7%) were females. As to the income, 103(25.5%) of the parents earned a mean monthly income of below 500 ETB (US$1 = 18.86 Ethiopian Birr). Regarding the educational status of the parents of the sampled children, 295(88.3%) of the fathers had formal education; 165(49.4%) had attended secondary school; 78(20.2%) mothers of the children were illiterate (Table 1). As to the occupation of the parents of the children, 140(34.7%) fathers were civil servants and 193(47.8%) mothers were housewives.

**Table 1:** Association between sociodemographic variables and anemia among school children, n = 404

**Sociodemographic variables**	**Total n(%) n = 404**	**Anemia**		**X**^**2**^	**p-value**
		**Present n(%) n = 152**	**Absent n(%) n = 252**		
**Sex**					
Male	187(46.3)	77(41.2)	110(58.8)	1.872	0.171
Female	217(53.7)	75(34.6)	142(65.4)		
**Age group**					
6**–**11 years	291(72.0)	118(40.5)	173(59.5)	3.796	0.51
12–14 years	113(28.0)	34(30.1)	79(69.9)		
**Religion**					
Muslim	140(34.7)	59(42.1)	81(57.9)		
Orthodox	199(49.3)	69(34.7)	130(65.3)	2.281	0.516
Protestant	61(15.1)	22(36.1)	39(63.9)		
Others*	4(0.01)	2(50.0)	2(50.0)		
**Ethnicity**					
Oromo	162(40.1)	72(44.4)	90(55.6)		
Amhara	87(21.5)	30(34.5)	57(65.5)		
Gurage	19(4.7)	8(42.1)	11(57.9)		
Kefficho	39(9.7)	10(25.6)	29(74.4)		
Dawro	39(9.7)	17(43.6)	22(56.4)	10.195	0.117
Yemme	21(5.2)	5(23.8)	16(76.2)		
Others**	37(9.2)	10(27.0)	27(73.0)		
**Marital status (Parents)**					
Living together	316(78.2)	112(35.4)	204(64.6)		
Divorced	13(3.2)	9(69.2)	4(30.8)	8.293	0.040
Widowed	52(12.9)	24(46.2)	28(53.8)		
Separated	23(5.7)	7(30.4)	16(69.6)		
**Family size**					
≤5	236(58.4)	86(36.4)	150(63.6)		
>5	168(41.6)	66(39.3)	102(60.7)	0.338	0.561
**Educational level (Fathers)**					
Illiterate	16(4.0)	10(62.5)	6(37.5)		
Read and write	23(5.7)	5(21.7)	18(78.3)	16.138	0.003
Primary level	91(22.5)	44(48.4)	47(51.6)		
Secondary level	165(40.8)	52(31.5)	113(68.5)		
Tertiary level	39(9.7)	10(25.6)	29(74.4)		
Missed	70(17.3)				
**Educational level (Mothers)**					
Illiterate	78(19.3)	46(59.0)	32(41.0)		
Read and write	42(10.4)	29(69.0)	13(31.0)	58.777	0.000
Primary level	105(26.0)	38(36.2)	67(63.8)		
Secondary level	139(34.4)	24(17.3)	115(82.7)		
Tertiary level	22(5.4)	6(27.3)	16(72.7)		
Missed	18(4.5)				
**Monthly income (ETB)**^**¥**^					
<500	103(25.5)	66(64.1)	37(35.9)		
500-999	139(34.4)	56(40.3)	83(59.7)	58.033	0.000
1000-1499	88(21.8)	17(19.3)	71(80.7)		
1500-1999	36(8.9)	9(25.0)	27(75.0)		
>2000	38(9.4)	4(10.5)	34(89.5)		
**Occupation (Fathers)**					
Farmer	32(7.9)	15(46.9)	17(53.1)		
Merchant	47(11.6)	7(14.9)	40(85.1)		
Civil servant	140(34.7)	53(37.9)	87(62.1)		
Hand craft	65(16.1)	18(27.7)	47(72.3)	22.113	0.000
Daily laborer	28(6.9)	17(60.7)	11(39.3)		
Others***	22(5.4)	11(50.0)	11(50.0)		
Missed	70(17.3)				
**Occupation (Mothers)**					
Merchant	78(19.3)	17(21.8)	61(78.2)		
Civil servant	69(17.1)	19(27.5)	50(72.5)		
House wife	193(47.8)	86(44.6)	107(53.4)		
Daily labor	20(5.0)	11(55.0)	9(45.0)	17.913	0.001
Others****	26(6.4)	10(38.5)	16(61.5)		
Missed	18(4.5)				

*Jehovah witness, Catholic. **Welita, Hadiya, Kembata, Silttia, Tigre, ^¥^US $1 = 18.86 Ethiopian Birr (ETB) according to the current exchange rate. ***Waiter, Factory worker, guard, retired. ****Waiter, Farmer, Factory worker, Hand craft, retired.

Concerning the results of the anthropometric measurements among anemic children, 13(8.6%) were thin, that is BMIFA < -2 z-score, and the prevalence of stunting that is height for age < -2 z-score was 12(7.9%).

### Prevalence of anemia

The study population was divided into anemic and non-anemic groups. The anemic group was further classified into mild, moderate and severe anemia. The proportion of children with anemia was 152(37.6%) as shown in Tables 1 and 2. The mean ± SD hemoglobin was 11.59 ± 1.97 g/dL. There was a significant difference in the mean ± SD distribution of Hb levels among the age group of 6–11 years (11.45 ± 2.08 g/dL) and age group of 12–14 years (11.93 ± 1.6 g/dL), p < 0.05. The mean ± SD value of hemoglobin in male children was 11.39 ± 1.94 g/dL, and in female children 11.76 ± 1.97 g/dL as shown in Table 2.

**Table 2:** Distribution of hemoglobin (Hb) level in different sex and age group among school children, n = 404

**Sex & age in years**	**Total N(%)**	**Hb (g/dL) Mean ± SD**	**t**	**CI (95 %)**	**p-value**
**Sex:**					
Male	187(46.3)	11.39 ± 1.94	1.89	-0.013, 0.75	0.058
Female	217(53.7)	11.76 ± 1.97			
**Age:**					
6-11	291(72)	11.45 ± 2.08	2.432	0.089, 0.855	0.016
12-14	113(28)	11.93 ± 1.60			
**Total**	404(100)	11.59 ± 1.97			

The prevalence of anemia among the age group of 6–11 years was 118(40.5%), while it was 34(30.1%) among the group of 12–14 years. The prevalence of anemia in children whose father’s are illiterate was 62.5%, and that of children whose mothers are illiterate was 59%. As per the monthly income of the families, the prevalence of anemia among children who belonged to families’ earning an average monthly income of less than 500 ETB (=US$26.5) was 64.1%. The highest prevalence (69.2%) of anemia was recorded in children whose parents were divorced. Therefore, the prevalence of anemia has a significant association with family income, parents’ educational status and marital status with p < 0.05 (Table 1).

### Severity of anemia

The grade of anemia was assessed by using the WHO’s cutoff values. Regarding the severity, among anemic children, 73/152(48.0%) of them had mild anemia while 79/152(52.0%) had moderate anemia. There was no single case of severe anemia observed in the present study.

### Association between anthropometric status, dietary factors and prevalence of anemia

According to the WHO reference standard, taking -2 SD (Z score) as a cutoff point [21], in this study children who fell below Z score of -2 SD of the indicators (stunted and thinness) were computed as 9.4% and 5.4%, respectively.

The occurrence of anemia was 59.1% in children whose BMI for age is below -2Z score (p = 0.033). There was no statistically significant association between anemia and height for age < -2Z score (Table 3).

**Table 3:** Anemia and its association with anthropometric measurements and dietary factors among school children, n = 404

**Variables**	**Total N(%)**	**Anemia**		**X**^**2**^	**p-value**	**COR**	**95% CI**
		**Absent N(%)**	**Present N(%)**				
**BMI for age < -2Z score**							
Yes	22(5.4)	9(3.6)	13(8.6)	4.569	0.033	0.39	0.16, 0.95
No	382(94.6)	243(96.4)	139(91.4)			1.00	
**Height for age < -2Z score**							
Yes	38(9.4)	26(10.3)	12(7.9)	0.653	0.419	1.34	0.65, 2.74
No	366(90.6)	226(89.7)	140(92.1)			1.00	
**Consumption of food from plant source**							
Less than once a day	186(46)	76(30.2)	110(73.4)	67.99	0.000	0.16	0.10, 0.25
Once a day or more	218(54)	176(69.8)	42(27.6)			1.00	
**Consumption of food from animal source**							
Less than once a week	304(75.2)	167(66.3)	137(90.1)	28.98	0.000	0.21	0.11, 0.38
Once a week or more	100(24.8)	85(33.7)	15(9.9)			1.00	

### Diet

About 54.0% of the study samples reported that they consumed plant food at least once a day; 24.8% of them consume animal food at least once a week. The occurrence rate of anemia was significantly higher in children who take foods of both plant and animal sources less frequently (p = 0.000) (Table 3).

### Correlates of anemia

To control the effect and predict the most important determinants of anemia, a stepwise logistic regression analysis was performed. In the model, the independent variables that had marginal and significant associations with anemia in the chi-square tests were computed. This model showed that mothers’ educational level, low family income, inadequate intake of plant and animal food were found to be significantly associated with anemia (Table 4).

**Table 4:** Multivariate logistic regression analysis showing predictors of anemia among school children, n = 404

**Variables**	**B**	**S.E**	**p-value**	**AOR**	**95% CI**
**Age**					
6**–**11 years	0.279	0.355	0.432	1.322	(0.659, 2.649)
12–14 years	0.0			1.00	
**Marital status (family)**					
Living together	0.0			1.00	
Widowed	0.583	0.573	0.102	1.791	(0.890, 3.604)
Separated	0.225	0.239	0.695	1.252	(0.407, 3.850)
**Educational level (Father)**					
Illiterate	1.026	0.955	0.283	2.790	(0.429, 18.122)
Read and write	-1.349	0.922	0.143	.260	(0.043, 1.580)
Primary level	0.020	0.643	0.976	1.020	(0.289, 3.593)
Secondary level	0.001	0.565	0.998	1.001	(0.331, 3.031)
Tertiary level	0.0			1.00	
**Educational level (Mother)**					
Illiterate	0.879	0.563	0.119	2.409	(0.799, 7.268)
Read and write	1.531	0.615	0.013	4.621	(1.383, 15.439)
Primary level	0.107	0.550	0.845	1.113	(0.379, 3.274)
Secondary level	-0.488	0.555	0.380	.614	(0.207, 1.824)
Tertiary level	0.0			1.00	
**Average monthly income (ETB)**^**¥**^					
<500	1.594	0.782	0.042	4.925	(1.063, 22.820)
500-999	1.230	0.695	0.077	3.421	(0.876, 13.361)
1000-1499	0.423	0.686	0.538	1.526	(0.398, 5.857)
1500-1999	0.645	0.761	0.397	1.905	(0.429, 8.461)
>2000	0.0			1.00	
**Occupation (Father)**					
Farmer	0.319	0.725	0.660	1.375	(0.332, 5.691)
Merchant	-0.090	0.794	0.910	.914	(0.193, 4.335)
Civil servant	0.323	.629	0.608	1.381	(0.403, 4.737)
Hand craft	-0.050	0.656	0.940	.952	(0.263, 3.445)
Daily labor	0.421	0.760	0.580	1.523	(0.344, 6.749)
Others***	0.0			1.00	
**Occupation (Mother)**		.			
Merchant	-0.192	848	0.820	.825	(0.157, 4.344)
Civil servant	0.747	0.857	0.383	2.112	(0.393, 11.335)
House wife	0.692	0.718	0.335	1.999	(0.489, 8.161)
Daily labor	1.127	1.088	0.300	3.087	(0.366, 26.017)
Others****	0.0			1.00	
**BMI for age < -2Z score**					
Yes	0.207	0.658	0.754	1.229	(0.338, 4.466)
No	0.0			1.00	
**Consumption of food from plant source**					
Less than once a day	1.347	0.317	0.000	3.847	(2.068, 7.157)
Once a day or more	0.00			1.00	
**Consumption of food from animal source**					
Less than once a week	863	420	0.040	2.370	(1.040, 5.402)
Once a week or more	0.0			1.00	

***Waiter, factory worker, guard, retired. ****Waiter, farmer, factory worker, hand craft, retired.

^¥^US $1 = 18.86 Ethiopian Birr (ETB) according to the current exchange rate.

## Discussion

The criteria for determining the presence of anemia, as recommended by the World Health Organization (WHO), are based on hemoglobin cutoff values for age and sex with an additional epidemiological criterion for assessing the severity and magnitude of the problem.

Measurement of hemoglobin level is a vital physiological parameter that helps diagnose the extent and severity of anemia, polycythemia as well as other diseases of red blood cells. The magnitude of anemia determined in this study (37.6%) is considered as a moderate public health problem according to World WHO standards [2].

The present finding is in agreement with those of related studies done in developing countries. The prevalence of anemia was 39.1% as it was done on 271 school-age children (age range: 7–14 years) in Asendabo Town, Southwest of Ethiopia [22]. It was 39.4% of 531 school-age children in Cote Divoire [23]; 36.9% in a group of 250 school-age children in Leyte, Philippines [24] and 36.4% among Vietnamese school age children [25] as determined by the same techniques. The result of this study also revealed that the prevalence rates of mild and moderate anemia were 18% and 19.6%, respectively. No single case of severe anemia (Hb < 7 g/dL) was detected in this study.

In the present study, mothers’ educational level and average monthly income were found to be important determinants of anemia. Low level of mothers’ education may affect children’s nutritional status negatively, and low income limits the type and amount of food available. In line with this finding, Mohamed *et al*. [26], Alemayehu [22], Jemal and Rebecca [27], Kaya *et al.*[28] and Bassam [29] reported that mothers’ educational level and low family income were found important determinants of anemia. Similarly, studies conducted on school-age children show the relationship between occurrence rate of anemia and family size [29] and anthropometric status of the children [29,30]. Additionally, father’s working status was found to be an important correlate of anemia as evidenced by studies done on 256 children (age range: 2 to 15 years) of Um-unnasser Village, Gaza [29] and in a group of 1295 school girls (age range: 6 to 18 years) in Ahmedabad city, Slums [30].

In the present study, about half of the children with anemia had lower intake of foods from animal sources, which is a source of heme iron. There are two forms of dietary iron: non-heme and heme iron. Non-heme iron takes the simplest form of free iron atoms such as ferric (Fe3+) or ferrous (Fe2+) iron. Non-heme iron is obtained from foods such as grains, pulses, legumes, fruits and vegetables [7]. In most populations throughout the world, non-heme iron is the main form of dietary iron. Heme iron is well absorbed in the intestine better than non-heme food obtained from plant sources. The finding of the present study indicated that more than half of the children who had anemia were eating food of plant source less frequently than non-anemic children. This dietary pattern would also result in low level of zinc and iron, which was found to be a strong predictor of hemoglobin level as studied in a group of 99 pregnant women [31] and in a group of 970 reproductive age women [27] in Ethiopia. A study conducted on pre-school children in Northern Ethiopia, Mekele, showed that the cultural food Teff-Enjera, which contains high amount of iron but bioavailability, was restricted because the type of iron is non-heme iron and there is an inadequate vitamin C, which reduces the absorption of iron [32]. Additionally, the major components of the diet in young children in developing countries are cereals and roots, which are not favorable for iron absorption as compared with meat and fish [33]. The present finding suggests high prevalence of anemia based on the fact that low level of Hb among school-age children probably due to dietary factors, inadequate consumption of animal food as mentioned above. Furthermore, reports of Djokic *et al.*[13] Kaya *et al.*[28] and Tiwari [34] show irregular consumption of meat and vegetable were found to be important correlates of anemia among school-age children. This fact supports the finding of this study.

Although not included in the present study, additional factors that may contribute to anemia in this population could be infection with intestinal parasites such as schistosomiasis, helminthes (hookworm in particular) and malaria.

## Conclusion

The prevalence of anemia among school-age children (6–14 years old) in Jimma Town was high (37.6%). This study also showed that mild and moderate levels of anemia were found to be 18% and 19.6% respectively, while no single case of severe anemia observed in the present study. It was established that occurrence of anemia is directly correlated with parents’ income and maternal literacy status. Children whose maternal education was limited to reading, and writing and children who were from families earning an average monthly income of less than 500 ETB (=US $26.5) were more likely to be affected by anemia. Participants who consumed animal and plant foods less frequently were more likely to develop anemia than that of more frequent users of these foods. This shows that the problem of anemia is linked with food insecurity because of economic constraints in the community.

Therefore, poverty alleviation and improving the economic status of the society is a crucial strategy to reduce the prevalence of anemia. General adult education to parents and health education to the community are also important strategies to reduce the burden of anemia. Consumer education is also recommended to encourage the use of diversified diets including iron-rich foods and fruits that contain vitamin C that enhances iron absorption. Additional studies are needed on micronutrients deficiency, parasite infections, hereditary disorders and environmental pollutants.

### Limitation of the study

This study has several limitations. It was done based on cross-sectional data, and we therefore, could not establish a cause effect relationship. We did a descriptive study that reports the prevalence and severity of anemia in general. The study lacks detailed investigation of the morphological appearance of red blood cells to differentiate anemia due to vitamin B_12_ and folic acid deficiencies from anemia due to iron deficiency. A study on the prevalence of iron deficiency anemia is being conducted by the team and will be reported soon. Stool test was not done to diagnose intestinal parasitic infection that contributes to anemia.

## Competing interests

The authors declare that they have no competing interests.

## Authors’ contributions

SA: involved in the preparation of the study design, participated in data collection, data entry and data analysis as well as manuscript preparation. AM: involved in the research proposal organization, data collection and data analysis and manuscript preparation. LH: involved in the preparation of the study design, data analysis and manuscript preparation. All authors read and approved the final draft of the manuscript.

## Pre-publication history

The pre-publication history for this paper can be accessed here:

http://www.biomedcentral.com/2052-1839/14/3/prepub
